# Oral human papilloma virus infection among dental clinic attendees in Ibadan, Nigeria

**DOI:** 10.4102/ajlm.v11i1.1555

**Published:** 2022-11-25

**Authors:** Adedayo O. Faneye, Oyeteju S. Babalola, Georgina N. Odaibo, Juwon Arotiba, Olufemi D. Olaleye

**Affiliations:** 1 Department of Virology, Faculty of Basic Medical Sciences, University of Ibadan, Ibadan, Nigeria; 2 Department of Oral and Maxilofacial Surgery, Faculty of Dentistry, University of Ibadan, Ibadan, Nigeria

**Keywords:** oral HPV infection, dental clinic attendees, molecular detection, HPV vaccine, Nigeria

## Abstract

**Background:**

Human papilloma virus (HPV) is associated with a subset of oropharyngeal squamous cell carcinoma and mouth or throat warts. However, there is currently limited information about oral HPV infections in Nigeria.

**Objective:**

This study aimed to provide information on the occurrence and circulating genotypes of HPV among patients attending three (one government and two private) dental clinics in Ibadan, Nigeria.

**Methods:**

An oral swab was collected from 231 dental clinic attendees in Ibadan between January 2016 and March 2017 and tested for HPV DNA by polymerase chain reaction targeting the E6/7 genes of the virus.

**Results:**

Twenty-three of the 231 swab samples were HPV DNA positive comprising 16 mono-infections and seven co-infections in 13 males and ten females. Genotype 16 was present in ten patients, genotype 6/11 in five, Genotype 18 and genotype 33 in four each, genotype 31 in three and genotype 39 in one. Twenty-one cases were high-risk HPV genotypes, while two were low-risk. Samples had co-infection and five had low risk type 6/11 either as single or as co-infection. Persons who had engaged in oral sex as well as those aged 21-30 years has significantly higher prevalence.

**Conclusion:**

This study showed that although HPV genotype 16 is the most common type among dental clinic attendees in Ibadan, other genotypes are also circulating and that oral sex is a risk factor for the infection. Therefore, introducing a multivalent HPV vaccine will reduce the risk of HPV-associated oropharyngeal carcinoma and other cancers in Nigeria.

## Introduction

Human papilloma viruses (HPV) are associated with anogenital cancer, including vulval, penile, vaginal, anal, and cervical cancer. These viruses have also been associated with a subset of head and neck cancers causing oropharyngeal squamous cell carcinoma.^1^ Apart from HPV genotypes associated with oropharyngeal carcinoma, infections with a few other genotypes of HPV can cause warts in the mouth or throat.^2^ Oral HPV infection is a common infection worldwide. A 4.9% oral HPV infection prevalence among healthy individuals worldwide has been reported. Southern Europe has the highest oral HPV prevalence (9.5%), while Central America has 6.6% and East Asia 0.6%. From the few studies in Africa, the prevalence of oral HPV ranges from 2.3% to 6.5%.^3^

The situation of HIV infection has caused an increase in the prevalence of oral HPV infection, especially among HIV-infected individuals in developed countries.^4^ However, information on the effect of HIV on the prevalence of oral HPV in Africa, and Nigeria in particular, is limited.

Human papilloma viruses are small, non-enveloped, double-stranded DNA viruses belonging to the Papovaviridae family. The family has five major genera: alpha, beta, gamma, mu, and nu, with those in the alpha genus having the most medical importance. Human papilloma viruses are further categorised into high risk or low risk based on their epidemiological associated malignancies.^5,6,7,8^ There are 14 HPV genotypes classified as high risk and they include HPV types 16, 18, 31, 33, 35, 39, 45, 51, 52, 56, 58, 59, 66 and 68, while genotypes 6, 11, 42, 43 and 44 are classified as low-risk types. Human papilloma virus infection of the mouth and the oropharynx, like HPV infection of the uterine cervix, is associated with high-risk sexual behaviours (orogenital sex). High-risk HPV genotypes, especially HPV-16, are present in many oral and oropharyngeal squamous cell carcinoma where, in some cases, they play an essential aetiological role.^7^

Presently, the prevalence of anogenital HPV infection in Africa ranges from 9.8% in Central Africa to 19.5% in Western Africa^9^; in Nigeria, it ranges from 3.5% to 28.8%. The common anogenital HPV genotypes circulating in Nigeria are HPV types 16, 18, 31, 35, 33, 39, 45, 51, 52, 56, 59, 70 and 82,^10^ while those associated with cancer in Nigeria include genotypes 16, 18, 33, 35, 45, 52 and 56.^10^ Although various HPV strains are circulating in Nigeria, the easily available vaccine is only against genotypes 16 and 18. Yet, the available HPV vaccine is not included in the national vaccination programme, and only a few informed individuals make private arrangements for the vaccination. The population of people making this arrangement is also low due to cost and awareness^11,12^; thus, the HPV vaccine coverage in Nigeria is meagre.

There is limited information on the prevalence and circulating genotypes of oral HPV in Africa and especially Nigeria. This information is essential to know if available vaccines will be effective in preventing oral HPV infection and associated oral cancers in Nigeria. This study was carried out to provide information on oral HPV and the circulating genotypes among patients presenting in dental clinics in Ibadan, Nigeria.

## Methods

### Ethical considerations

This study was approved by the Ethics Review Committee of the University of Ibadan and University College Hospital Ibadan with approval number 160057, and participants provided written informed consent. Confidentiality of the study participants was maintained; participants’ identities were codified, and only the authorised party could link the identity of the participants to the given code when the result is given to the dentist to notify the participants of their result.

### Study design

This study was a descriptive cross-sectional study carried out among 231 patients attending three dental clinics comprising one government (University College Hospital Ibadan Dental Clinic) and two private dental clinics in Ibadan, Oyo State, Nigeria, from August 2016 to February 2017. Government dental clinics in Ibadan provide dental services for the population of Ibadan and its environment. The minimum sample size calculated was 196 but a total of 231 individuals was recruited for the study. The sample size was determined using the formula:


$$N=Za^{2}pQ/d^{2}$$ [Eqn 1]

a prevalence of 15%^13^ at 95% confidence interval and 0.02 level of variability of the target population (*N* = the sample size, *a* = 1.96, *p* = prevalence, *Q* = 1 – *p*, and *d* = 0.05).

### Study population

A total of 231 individuals who consented and were aged 18 years or older were recruited for the study. The participants were first-time attendees of dental clinics (one public and two private) in Ibadan, Oyo State, Nigeria, presenting with various dental complaints. A convenient sampling method was used.^14^ Individuals were excluded if they were unable or unwilling to provide consent or were in severe pain or with symptoms of oropharyngeal cancer.

### Sample collection and processing

Socio-demographic and risk behaviour information (presence of oral warts, smoking, number of sexual partners, previous oral infection, and engaging in oral sex) was collected from each participant using a semi-structured questionnaire. A dental technician trained in collecting oral swabs for HPV testing collected oral swab samples from all participants. Briefly, the tip of the swab stick was rubbed several times in the mouth and base of the tongue. The swab sticks were then placed in vials containing 500 µL of transport medium containing minimum essential medium and 2% bovine serum albumin with antibiotics (Gentamicin) in a secured capped vial. Samples were stored and transported at 4 °C to the laboratory. Initial processing in the laboratory was done within 24 h of sample collection, and included vortexing each vial containing transport medium, removing the swab; the medium was aliquoted and stored at –80 °C until analysed for HPV DNA by polymerase chain reaction (PCR).

### Laboratory analysis

Total DNA was extracted from each sample using a commercially available DNA purification kit (Jena Bioscience, Jena, Germany) per the manufacturer’s instruction. The extracted DNA was tested for the presence of the E6/E7 HPV viral gene by PCR using previously described primers (GP E6-3F, GP-E6-5B and GP-E6-6B) and protocol.^15^ Amplification was achieved using a ABI 9700 GeneAmp thermal cycler (Applied Biosystems^®^, Waltham, Massachusetts, United States) using the following cycling condition: 5 min at 95 °C for DNA denaturation followed by 65 cycles of 30 s of denaturation at 95 °C, 30 s of annealing at 45 °C, 30 s of elongation at 68 °C and a final elongation of 5 min at 72 °C. The amplified products (602 base pairs – 666 base pairs) were detected using agarose gel electrophoresis. Human papilloma viruses isolates were typed using genotype specific primers targeting the E6/E7 HPV virus gene as previously described^15^ (Table 1). The primers used could not differentiate between genotypes 6 and 11, thus, it is referred to as genotype 6/11.

**TABLE 1:** List of primres used and the amplicon size.

No.	Target	Primer name	Sequence	Amplicon size (base pairs)
1	E6/& consensus	GP-E6-3F	GGG WGK KAC TGA AAT CGG T	602–666
		GP-E6-5b	CTG AGC TGT CAR NTA ATT GCT CA	
		GP-E7-6B	TCC TCT GAG TYG YCT AAT TGC TC	
2	HPV genotype 16	HPV16-F	CAC AGT TAT GCA CAG AGC TGC	457
		HPV16-R	CAT ATA TTC ATG CAA TGT AGG TGT A	
3	Genotype 18	HP18-F	CAC TTC ACT GCA AGA CAT AGA	322
		HPV-18-R	GTT GTG AAA TCG TCG TTT TTC A	
4	HPV genotype 31	HPV31-F	GAA ATT GCA TGA ACT AAG CTC G	263
		HPV31-R	CAC ATA TAC CTT TGT TTG TCA A	
5	HPV genotype 33	HPV33-F	ACT ATA CAC AAC ATT GAA CTA	398
		HPV33-R	GTT TTT ACA CGT CAC AGT GCA	
6	HPV genotype 35	HPV35-F	CAA CGA GGT AGA AGA AAG CAT C	358
		HPV35-R	CCG ACC TGT CCA CCG TCC ACC G	
7	HPV genotype 39	HPV39-F	GAC GAC CAC TAC AGC AAA CC	280
		HPV39-R	TTA TGA AAT CTT CGT TTG CT	
8	HPV genotype 42	HPV42-F	CCC AAA GTA GTG GTC CCA GTT A	277
		HPV42-R	GAT CTT TCG TAG TGT CGC AGT G	
9	HPV genotype 43	HPV43-F	GCA TAA TGT CTG CAC GTA GCT G	219
		HPV43-R	CAT GAA ACT GTA GAC AGG CCA AG	
10	HPV genotype 44	HPV44-F	TAA ACA GTT ATA TGT AGT GTA CCG	163
		HPV44-R	TAT CAG CAC GTC CAG AAT TGA C	
11	HPV genotype 45	HPV45-F	GTG GAA AAG TGC ATT ACA GG	151
		HPV45-R	ACC TCT GTG CGT TCC AAT GT	
12	HPV genotype 51	HPV51-F	GAG TAT AGA CGT TAT AGC AGG	233
		HPV51-R	TTT CGT TAC GTT GTC GTG TAC G	
13	HPV genotype 52	HPV52-F	TAA GGC TGC AGT GTG TGC AG	229
		HPV52-R	CTA ATA GTT ATT TCA CTT AAT GGT	
14	HPV genotype 56	HPV56-F	GTG TGC AGA GTA TGT TTA TTG	181
		HPV56-R	TTT CTG TCA CAA TGC AAT TGC	
15	HPV genotype 58	HPV58-F	GTA AAG TGT GCT TAC GAT TGC	274
		HPV58-R	GTT GTT ACA GGT TAC ACT TGT	
16	HPV genotype 59	HPV59-F	CAA AGG GGA ACT GCA AGA AAG	215
		HPV59-R	TAT AAC AGC GTA TCA GCA GC	
17	HPV genotype 66	HPV66-F	TTC AGT GTA TGG GGC AAC AT	172
		HPV66-R	AAA CAT GAC CCG GTC CAT GC	
18	HPV genotype 68	HPV68-F	GCA GAA GGC AAC TAC AAC GG	333
		HPV68-R	GTT TAC TGG TCC AGC AGT GG	

Note: The three primers were used together and the amplicon size ranges from 602-666.

HPV, human papilloma virus.

### Data analysis

The data anlalysis was done using Statistical Package for Social Sciences (SPSS) version 18.2 (IBM Corp. Armonk, New York, United States). All the data generated were analysed using descriptive statistics such as mean and standard deviation and results were analysed using chi-square at α = 0.05.

## Results

### Characteristics of the study population

A total of 231 study participants were recruited for this study, out of which 129 (55.9%) were female. Their mean age was 42.59 (range: 18–62 years) (Table 2). All the participants reported having sexual experience, while 46 (20.0%) had engaged in oral sex. Thirty-six (15.6%) of the participants had warts, either oral or genital, while 24 (10.4%) were smokers. Fifty-four (23.4%) of the participants had more than one sexual partner, and none had received any HPV vaccination.

**TABLE 2:** Age distribution of dental clinic attendees in Ibadan, Nigeria, January 2016 to March 2017.

Category	Total
**Age (years)**	
< 20	18
21–30	55
31–40	42
41–50	37
51–60	32
> 60	47
**Gender**	
Male	104
Female	127
**Marital status**	
Married	128
Single	90
Widowed	2
Divorced	11

Note: Mean age = 42.59, standard deviation = 15.85, variance = 251.32.

### Human papilloma virus DNA prevalence and genotypes

Of the 231 oral swabs tested, 23 (9.9%) had HPV DNA, of which 21 were high-risk and two were low-risk HPV. Of the 23 HPV DNA-positive participants, 13 (56.5%) were male and 10 (43.5%) were female. Also, 18 (78.3%) of the 23 participants with HPV had previously engaged in oral sex. Participants in the age group 21–40 years had the highest rate of positivity (14.5%), whereas those aged 60 years or older had the lowest rate (2.1%) (*p* = 0.241) (Table 3).

**TABLE 3:** Distribution of human papilloma virus infection among dental clinic attendees in Ibadan, Nigeria, January 2016 to March 2017.

Variable	Category	Number tested	HPV DNA positive	%	Chi-square	*p*
Presence of oral warts					1.0629	0.303
	Yes	36	2	5.6	-	-
	No	168	19	11.3	-	-
Smoking					1.860	0.173
	Yes	24	4	16.7	-	-
	No	207	17	8.2	-	-
Number of sexual partners					4.208	0.121
	1	146	11	7.5	-	-
	> 1	54	9	16.7	-	-
	None	31	1	3.2	-	-
Previous oral infection					0.696	0.404
	Yes	108	8	7.4	-	-
	No	123	13	10.6	-	-
Gender					1.371	0.242
	Male	104	12	11.5	-	-
	Female	127	9	7.1	-	-
Marital status					1.896	0.425
	Married	128	9	7.0	-	-
	Single	90	11	12.2	-	-
	Widowed	2	0	0.0	-	-
	Divorced	11	1	9.1	-	-
Ever engaged in oral sex					62.715	0.0001
	Yes	46	18	39.1	-	-
	No	185	3	1.6	-	-
Age					134.860	0.0001
	18–20	18	1	5.6	-	-
	21–40	97	14	14.4	-	-
	41–50	69	5	7.2	-	-
	≥ 60	47	1	2.1	-	-

Of the 23 participants with HPV infection, 16 had mono infections while seven had co-infections with either other high-risk or low-risk genotypes. The most common genotypes detected in this study were genotypes 16 and 18. HPV genotype 16 was detected in ten participants, eight of which were single infection and one co-infection with genotype 33, the other one were the low risk group (6/11). Genotype 18 was also detected in four participants, of which three were single infection and one co-infection with genotype 31. Genotypes 31 and 33 were each detected in three participants, two as single infection and one each as co-infection with other genotype. Genotype 39 was the least prevalent genotype detected in only one participants. A total of 21 participants had high-risk HPV DNA.

## Discussion

The prevalence of oral HPV infection and the circulating genotypes among dental clinic attendees in Ibadan, Nigeria, was determined in this study. An oral HPV prevalence of 9.9% was observed. Previous reports on the rates of oral HPV infection among asymptomatic individuals varied between different geographic areas. It ranged from 12.0% in South Africa to 5.0% in the United States. The prevalence obtained among dental clinic attendees in this study is lower than the 12% reported from South Africa among individuals attending HIV testing centres^13^ and higher than the 7.3% and 6.9% reported from the general population in the United States.^16,17^ The differences obtained could be due to the population tested. The higher prevalence in the South African population could be due to the underlying HIV infection. It was also noted that the prevalence of oral HPV among HIV-positive individuals in the United States is also high.

The primers used for the detection of HPV DNA in this study target the E6/7 region of the virus. This region is part of the viral oncogenes and is usually integrated into the host genome. Thus, primers targeting this region are more sensitive than the L1 region most commonly used. Other studies have shown that PCR protocols targeting the L1 region of the HPV genome are likely to miss some infections if the viral DNA has been integrated, as the L1 region of the viral genome is usually deleted during viral integration.^18,19^ In addition, the detection of the E6/7 genes of the HPV can suggest a persistent HPV infection, which could be used to predict people at risk of oropharyngeal carcinoma.

In this study, people aged 60 years and older had the lowest prevalence (2.1%) of HPV infection, while those in the age group of 21–40 years had the highest rate (14.5%) (Table 3). The 21–40 age group is the most sexually active age group; thus, the pattern may result from sexual activities and engagement in oral sex among the persons within this age range. Studies have shown that this is the age group most likely to engage in oral sex. In addition, oral sex was not as common in the past as it is now. This may be why the prevalence of oral HPV infection is higher among younger participants. The age distribution found in this study is similar to what was reported by Antonsson et al.^20^ in Australia but different from the pattern reported in the United States by Sanders et al.,^16^ where the highest prevalence (11.3%) was observed among the age group 55–64 years.

Gender differences have been observed in the distribution of oral HPV.^8,9^ In this study, there are no significant differences in the gender distribution of HPV DNA.^21,20^ Furthermore, findings from this study showed that participants with more than one sexual partner had a higher prevalence of oral HPV infection. Positive oral HPV testing is significantly positively correlated with an increase in vaginal sexual partners.^21^ There was also no significant difference in prevalence of HPV DNA as per marital status of the participants in this study, although Kero et al.^22^ reported that stable marital relationships protect men from oral and genital HPV infection. This study also noted that participants who engaged in oral sex had a significantly higher prevalence of oral HPV infection. Oral sex is a major route for HPV transmission.^21,23,24^ Although oral sex and having multiple sexual partners are shown to be significantly associated with oral HPV positivity, other behaviours such as kissing can transmit HPV.^25^

This study detected low risk HPV genotype 6/11 and high-risk genotypes 16, 18, 31, 33 and 39 among dental clinic attendees in Oyo state, Nigeria. Among the previously reported circulating genital HPV types among women in Ibadan, Nigeria, according to Nejo et al. and Thomas et al.^26,27^ The most common HPV genotype (type 16) in this study is similar to the findings of Thomas et al. but different from the report of Nejo et al., which reported HPV genotype 31 as the most prevalent. Human papilloma viruses genotype 16 has also been reported from other parts of the world as the most common oral HPV type.

Finally, the inclusion of the HPV vaccine into the national vaccination programme to cover both boys and girls in Nigeria will reduce the prevalence of oral HPV infection and, ultimately, HPV-associated oropharyngeal cancer in Nigeria. Presently, the most common vaccine in Nigeria (Cervarix marketed by GlaxoSmithKline) targets HPV 16 and 18 and Gardasil (marketed by Merk & Co.) targets only HPV 16, 18, 6 and 11 whereas this study and previous Nigerian studies have identified other genotypes. Thus, introducing HPV vaccine that will cover other circulating strains in addition to the ones they cover presently will make the vaccination programme more effective. The study by Herrero et al.^28^ has demonstrated the efficacy of HPV vaccination in reducing prevalence of oral HPV after four years of vaccinating women in Costa Rica.

### Limitations

Patients with symptoms of oropharyngeal cancer were not included in this study to identify the virus genotypes associated with oropharyngeal cancer in this region. Information about the number of kissing partners of the participants was also not accessed in the study. The size of the study population is also a limitation of the study.

### Conclusion

This study describes a high prevalence of oral HPV infection among dental clinic attendees in Ibadan, Oyo State, Nigeria, by detecting HPV DNA from the oral swabs of the study participants using PCR. A prevalence of 9.9% of HPV infection was identified and also showed that HPV type 16 and 18 are the most common types detected among the study participants. Oral sex was also significantly associated with HPV infection. The HPV vaccine for use in Nigeria should cover at least the commonest circulating genotypes to reduce the risk of HPV-associated oropharyngeal and other cancers in Nigeria.
